# Changes in spending, utilization, and quality of care among Medicare accountable care organizations during the COVID-19 pandemic

**DOI:** 10.1371/journal.pone.0272706

**Published:** 2022-08-12

**Authors:** Brandon W. Yan, Maya Shashoua, Jose F. Figueroa

**Affiliations:** 1 Department of Health Policy and Management, Harvard T. H. Chan School of Public Health, Boston, Massachusetts, United States of America; 2 School of Medicine, University of California San Francisco, San Francisco, California, United States of America; 3 Department of Medicine, Harvard Medical School, Boston, Massachusetts, United States of America; Universidad Nacional Autonoma de Nicaragua Leon, NICARAGUA

## Abstract

The COVID pandemic disrupted health care spending and utilization, and the Medicare Shared Savings Program (MSSP), Medicare’s largest value-based payment model with 11.2 million assigned beneficiaries, was no exception. Despite COVID, the 513 accountable care organizations (ACO) in MSSP returned a program record $1.9 billion in net savings to Medicare in 2020. To understand the extent of COVID’s impact on MSSP cost and quality, we describe how ACO spending changed in 2020 and further analyze changes in measured quality and utilization. We found that non-COVID per capita spending in MSSP fell by 8.3 percent from $11,496 to $10,537 (95% confidence interval(CI),-1,223.8 to-695.4, p<0.001), driven by 14.6% and 7.5% reductions in per capita acute inpatient and outpatient spending, respectively. Utilization fell across inpatient, emergency, and outpatient settings. On quality metrics, preventive screening rates remained stable or improved, while control of diabetes and blood pressure worsened. Large reductions in non-COVID utilization helped ACOs succeed financially in 2020, but worsening chronic disease measures are concerning. The appropriateness of the benchmark methodology and exclusion of COVID-related spending, especially as the virus approaches endemicity, should be revisited to ensure bonus payments reflect advances in care delivery and health outcomes rather than COVID-related shifts in spending and utilization patterns.

## Introduction

The COVID pandemic has led to substantial disruptions across the US health care system, and the Medicare Shared Savings Program (MSSP), Medicare’s largest value-based payment model, was no exception. Early in the pandemic, the majority of Accountable Care Organizations (ACOs) expressed major concerns that the pandemic would derail their efforts to achieve high performance and meaningful savings[1]. However, in 2020, the 513 ACOs in MSSP caring for 11.2 million beneficiaries returned a program record $1.9 billion (or $190 per beneficiary) in net savings to Medicare compared to the benchmark. This occurred despite tremendous uncertainty about health care costs introduced by the COVID-19 pandemic [1, 2].

Enacted in statute by the Affordable Care Act, MSSP began in 2012 as part of federal efforts to reduce health care spending while maintaining or improving quality of care. Since then, the ACOs in MSSP have returned modest but gradually increasing net savings to the Centers for Medicare & Medicaid Services (CMS) and demonstrated ability to meet quality benchmarks [3–6]. However, the assessment of financial savings remains controversial as they reflect the difference between what Medicare expected to pay for care (i.e., the cost benchmark) and what actually transpired in Medicare claims and, therefore, lacks a true control group from which to assess spending in the absence of the program [7, 8]. Still, the general consensus is that the program achieves some savings with potential for further growth and savings with programmatic changes [3, 7–10].

As the COVID-19 pandemic began, there were concerns that a large portion of ACOs would likely exit the program due to concerns that that they would fare poorly [1]. In response, CMS introduced program flexibilities that excluded COVID care from counting against an ACO’s cost benchmark and reduced the number of quality metrics from which quality performance was assessed among other changes [3, 11, 12]. While prior work has assessed overall MSSP performance on cost and quality against benchmarks [3], detailed analysis by individual expenditure categories, service utilization, and quality measures have not been published previously. Therefore, in our study, we compare MSSP performance on cost, utilization, and quality in 2020 to 2019 at the aggregate and individual levels to elucidate where cost savings were derived from, what services were less utilized, and where quality of care improved or deteriorated. These insights offer a more in-depth understanding of program performance during a pandemic and areas of policy concern for future years.

## Study data & methods

We performed an observational cohort study of ACO performance in MSSP. Using data from the MSSP Public Use Files (PUF), we examined the ACO program’s cost performance and service utilization rates in 2020 compared to 2019. The PUF is a program dataset released by CMS for each performance year that contains data on each participating ACO’s beneficiary spending by service line, service utilization rates, and quality ratings.

First, we compared yearly total Part A and B per capita spending in 2020 versus 2019. We calculated a change in per capita spending as well as a percent change. Data in the PUF excluded COVID-related costs since these costs were excluded from the ACO benchmark as part of pandemic flexibilities introduced by CMS. We also calculated an annual change in total per capita spending between 2017 and 2019 as a baseline trend with which to compare the 2019 to 2020 change to assess whether the latter is a novel change or continuation of existing trends.

Second, we examined changes in spending by three care settings: acute inpatient, total outpatient, and post-acute care. These broad categories were created by aggregating per capita spending in the following service lines provided in the PUF: acute inpatient care included short term acute care hospital, inpatient psychiatric hospital, and other inpatient service; total outpatient included outpatient and physician/supplier services; post-acute care included expenditures for long term care hospital (LTCH), inpatient rehabilitation facility (IRF), hospice, skilled nursing facility/unit (SNF), and home health services. The calculations performed for total Part A and Part B spending were repeated for each expenditure category and subcategory for two reasons: 1) To determine which service lines drove the change in overall spending, and 2) To derive potential insights into where and how the COVID pandemic may have affected spending.

Third, utilization was assessed using the aforementioned calculations for the following metrics available in the PUF: Inpatient Hospital Discharges (ADM), short term acute care hospital discharges, long term care hospital discharges, inpatient rehabilitation facility discharges, inpatient psychiatric facility discharges, congestive heart failure discharges, chronic obstructive pulmonary disease (COPD) or asthma discharges, post-discharge provider visits within 30 days, outpatient emergency department (ED) visits, inpatient ED visits, computerized topography (CT) events, magnetic resonance imaging (MRI) events, total primary care services, primary care services with a primary care physician (PCP), primary care services with a specialist, primary care services with a nurse practitioner (NP)/physician assistant (PA)/clinical nurse specialist (CNS), primary care services with a federally qualified health center (FQHC) or rural health clinic (RHC), and skilled nursing facility discharges.

Fourth, we analyzed changes in quality performance on the total quality score and 11 measures assessed in the 2020 performance year: screening for fall risk, influenza immunization, tobacco use screening and cessation intervention, screening for depression and follow-up plan, colorectal cancer screening, breast cancer screening, diabetes control (defined as hemoglobin A1c higher than 9%), blood pressure control (defined as less than 140/90 mmHg), depression remission at 12 months, statin therapy for prevention and treatment of cardiovascular disease (CVD), and ambulatory sensitive condition acute composite (ASCAC) per 100 person years.

The total quality score comparison between 2020 and 2019 is complicated by benchmark flexibilities introduced by CMS that awarded automatic full credit for the 10 Consumer Assessment of Healthcare Providers and Systems (CAHPS) measures in the patient/caregiver domain and reversion of two other quality measures, risk-standardized all condition readmissions and all-cause unplanned admissions for patients with multiple chronic conditions, to pay-for-reporting. Therefore, whereas overall ACO quality in 2019 was assessed on 23 measures, the 2020 value is based on 11 performance and 2 reporting measures.

### Statistical analysis

The average value in each of the cost, utilization, and quality metrics for 2020 was compared to the average value in 2019 using the two-sided t-test. Since ACOs participated for either 6 months or 12 months in 2019 due to the coexistence of legacy and Pathways to Success ACO contracts, analyses weighted performance based on ACO contract length in 2019 (0.5 if half-year contract, 1 if full-year contract). Statistical significance was defined at p-values of less than 0.05. All analyses were conducted using STATA Version 15.1 (College Station, TX).

### Sensitivity analysis

Due to annual churn in terms of ACOs entering and leaving MSSP, we conducted a sensitivity analysis wherein the analyses described above were repeated for just those ACOs with contracts in both 2019 and 2020. The rationale is to demonstrate that differences in average program differences between the two performance years in the main analysis were due to changes in the ACOs themselves and not due to year-to-year selection bias.

### Limitations

Our study has important limitations. First, given that this is an observational study, we cannot definitively conclude that the changes in MSSP performance between 2019 and 2020 are due to a causal effect of MSSP itself, disruptions in care resulting from the COVID-19 pandemic, or due to other broader market changes[13]. Second, MSSP is voluntary, which exposes the program to selection bias and the possibility that the ACO providers in 2020 were different from those in 2019 in ways that enabled them to succeed during COVID, although our sensitivity analyses suggest that this was not the case. The introduction of Pathways to Success in July 2019 with simultaneous operation of legacy program contracts complicates the use of 2019 as a baseline year. These program realities preclude a more perfect analysis. However, our concern of selection bias was addressed through sensitivity analysis restricting the sample to only those ACOs that operated in both performance years. Third, as described previously, total quality score between 2019 and 2020 are difficult to compare due to differences in metrics used for calculation. Still, they offer the best available aggregate assessment of quality available for each performance year. Another limitation is that the publicly-available file presents aggregated data on spending and utilization, and thus we were unable to risk-adjust at the individual beneficiary level when examining changes over time. Prior work, however, has found that the characteristics of patients enrolled in ACOs has not changed significantly over time and thus is unlikely to change our findings [14]. Lastly, we do not consider the effects of a changing climate (seasonal or long-term) that may be a source of variation in COVID-affected healthcare spending as it pertains to 2019 and 2020 spending [15, 16].

## Results

### Changes in spending

A total of 680 ACO contracts (475 legacy program and 205 Pathways to Success among 541 unique ACO entities) in 2019 and 513 in 2020 were included for analysis, along with 472 and 548 in 2017 and 2018, respectively, for comparison.

**Fig 1:**
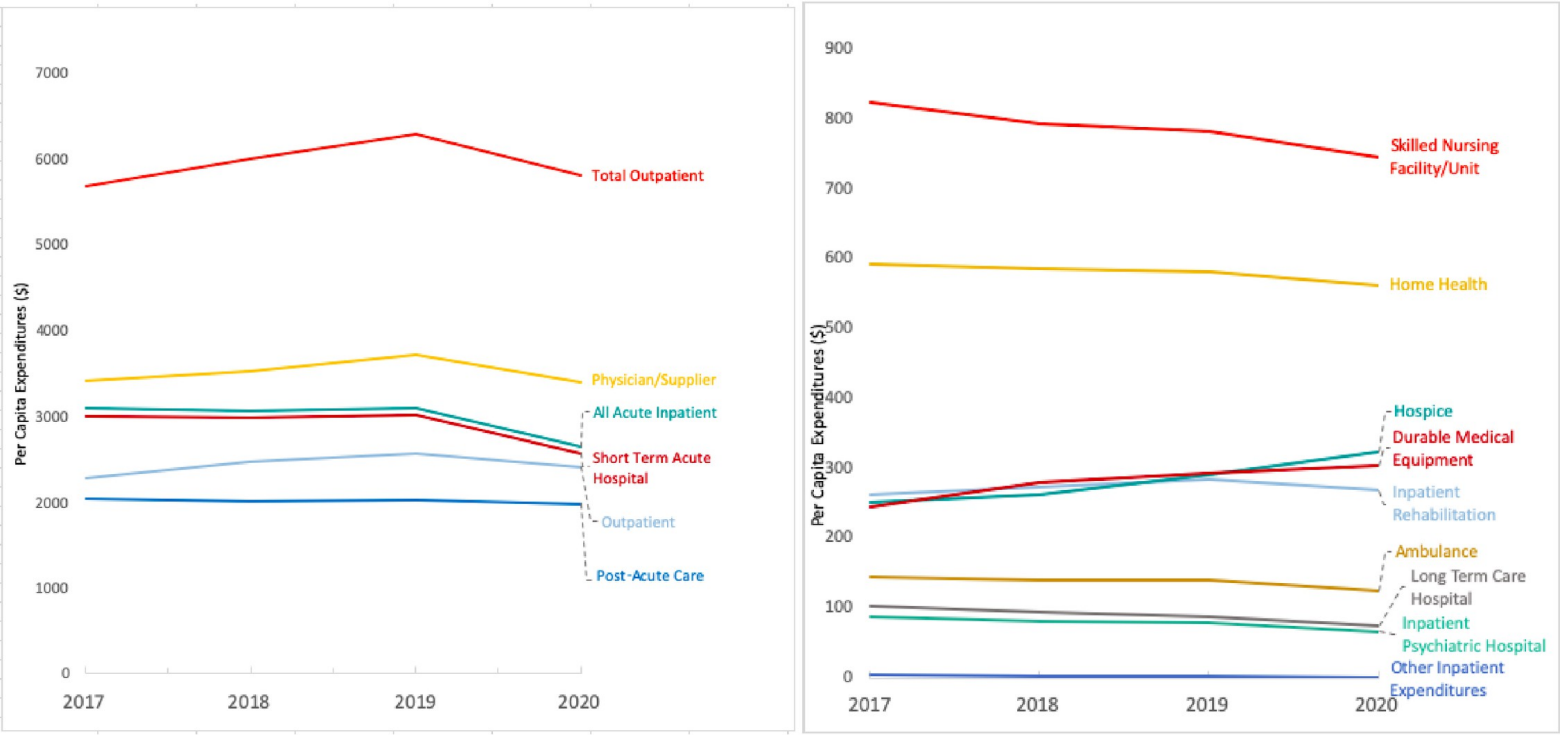
a) Changes in MSSP per capita expenditures by service line above $1,000, 2017–20, b) Changes in MSSP per capita expenditures by service line below $1,000, 2017–20.

Between 2019 and 2020, total per capita spending in MSSP fell 8.3 percent from $11,496 to $10,537 (95%CI, -1223.8 to -695.4, p<0.001) (Fig 1 and Table 1). All service lines saw declines in per capita spending except hospice (+11.1%) and durable medical equipment (+3.4%) (Table 1). Acute inpatient spending per capita experienced the largest decline at 14.6% (95%CI, -532.0 to -366.4, p<0.001) followed by a 7.5% drop in total outpatient spending (95%CI, -574.4 to -373.7, p<0.001). Post-acute care spending did not change statistically (95%CI, -192.5 to 86.1, p = 0.45), although the subcategory of long-term care hospital spending saw a 14.1% drop (95%CI, -23.4 to -4.8, p = 0.003) (Table 1).

**Table 1:** Changes in per capita expenditures by service line, 2017–2020.

Per Capita Expenditure	2017, $	2019, $	2020, $	Annual Change, 2017–19	Annual Change 2019–20		
					$ (% Change)	95% CI	p value
	(n = 472)	(n = 680)	(n = 513)	$ (% Change)			
Total	10865.4	11496.1	10536.5	315.3 (2.9)	-959.6 (-8.3)	-1223.8 to -695.4	<0.001
**Acute Inpatient Care**	**3094.1**	**3086.9**	**2637.7**	**-3.6 (-0.1)**	**-449.2 (-14.6)**	**-532.0 to -366.4**	**<0.001**
Short Term Acute Care Hospital	3001.4	3017.2	2570.7	7.9 (0.3)	-446.5 (-14.8)	-520.2 to -372.9	<0.001
Inpatient Psychiatric Hospital	88.8	78.2	67.0	-5.3 (-6.0)	-11.2 (-14.3)	-19.3 to -3.0	0.007
Other Inpatient^a^	3.9	3.7	--	-0.1 (-2.5)	--	--	--
**Post-Acute Care**	**2033.4**	**2027.0**	**1973.8**	**-3.2 (-0.2)**	**-53.2 (-2.6)**	**-192.5 to 86.1**	**0.454**
Long Term Care Hospital	102.8	88.7	74.6	-7.0 (-6.9)	-14.1 (-15.9)	-23.4 to -4.8	0.003
Inpatient Rehabilitation Facility	262.0	284.4	268.3	11.2 (4.3)	-16.2 (-5.7)	-3.8 to 2.5	0.090
Hospice	252.2	290.3	322.6	19.0 (7.5)	32.3 (11.1)	-8.1 to 72.8	0.117
Skilled Nursing Facility or Unit	823.2	781.7	745.7	-20.7 (-2.5)	-36.0 (-4.6)	-115.0 to 42.7	0.369
Home Health	593.2	581.8	562.5	-5.7 (-1.0)	-19.3 (-3.3)	-58.2 to 19.6	0.331
**Total Outpatient**	**5692.0**	**6284.7**	**5810.7**	**296.3 (5.2)**	**-474.0 (-7.5)**	**-574.4 to -373.7**	**<0.001**
Outpatient	2277.3	2567.7	2409.8	145.2 (6.4)	-157.9 (-6.1)	-249.8 to -65.8	0.001
Physician/Supplier	3414.8	3717.0	3400.8	151.1 (4.4)	-316.2 (-8.5)	419.2 to -213.2	<0.001
Durable Medical Equipment	244.0	292.9	303.0	24.4 (10.0)	10.1 (3.4)	1.8 to 18.7	0.017
Ambulance	144.7	139.9	124.6	-2.4 (-1.6)	-15.3 (-10.9)	-24.1 to -6.5	0.001

^a^ The other inpatient spending measure was not reported for 2020.

Within the aggregate acute inpatient spending category, both short-term acute care hospital (-14.8%, 95%CI, -520.2 to -372.9, p<0.001) and inpatient psychiatric (-14.3%, 95%CI, -19.3 to -3.0, p = 0.007) per capita spending fell. However, the decrease in short-term acute care hospital spending drove the aggregate decline, as inpatient psychiatric services constituted just 2.5% of acute inpatient spending in 2020 (Table 1). Furthermore, per capita inpatient psychiatric spending was downtrending even prior to 2020 (Fig 1 and Table 1).

### Changes in utilization

The decline in non-COVID related spending paralleled declines across most utilization measures (Table 2). Inpatient hospital discharges per 1,000 person years fell 13.6% (p<0.001) from 2019 to 2020 with COPD and asthma discharges seeing the largest decline at 42.3% fewer discharges per 1,000 person years (p<0.001) (Table 2). Emergency department (ED) visits declined more than 10 percent across inpatient and outpatient settings, while CT and MRI utilization per 1,000 person years fell 8.3% (p<0.001) and 11.2% (p<0.001), respectively (Table 2).

**Table 2:** Changes in utilization, 2017–2020.

Utilization Measure (per 1000 person years):	2017 (n = 472)	2019 (n = 680)	2020 (n = 513)	Annual Change 2017–2019 (% Change)	Annual Change 2019–20		
					Annual Change (% Change)	95% CI	p value
Inpatient hospital discharges	324.9	310.5	268.2	-7.2 (-2.2)	-42.3 (-13.6)	-49.6 to -34.9	<0.001
Short term acute care hospital discharges	299.2	286.0	246.8	-6.6 (-2.2)	-39.2 (-13.7)	-45.9 to -32.5	<0.001
Long term care hospital discharges	3.1	2.4	2.2	-0.4 (-11.3)	-0.2 (-8.3)	-0.5 to 0.1	0.187
Inpatient rehab facility discharges	13.5	14.2	13.0	0.4 (2.6)	-1.2 (-8.5)	-2.2 to -0.3	0.012
Inpatient psychiatric facility discharges	8.9	7.6	6.0	-0.7 (-7.3)	-1.6 (-21.1)	-2.2 to -0.9	<0.001
Congestive heart failure discharges	16.7	16.7	13.5	0.0 (0.0)	-3.2 (-19.2)	-3.6 to -2.6	<0.001
COPD or asthma discharges	12.0	7.8	4.5	-2.1 (-17.5)	-3.3 (-42.3)	-3.6 to -3.0	<0.001
Post-discharge provider visits (30 day)	805.8	809.3	779.9	1.8 (0.2)	-29.4 (-3.6)	-33.3 to -25.4	<0.001
Outpatient ED visits	742.6	710.5	579.5	-16.1 (-2.2)	-131.0 (-18.4)	-147.4 to -114.6	<0.001
Inpatient ED visits	218.9	215.9	192.7	-1.5 (-0.7)	-23.2 (-10.7)	-30.3 to -16.1	<0.001
CT events	819.9	707.4	648.5	-56.3 (-6.9)	-58.9 (-8.3)	-71.4 to -46.4	<0.001
MRI events	301.3	245.7	218.1	-27.8 (-9.2)	-27.6 (-11.2)	-33.7 to -21.5	<0.001
Primary care services	10454	10971	10234	258.3 (2.5)	-736.6 (-6.7)	-1013.6 to -459.8	<0.001
Primary care services with PCP	4110.7	4212.2	3933.8	50.8 (1.2)	-278.4 (-6.6)	-463.4 to -93.3	0.003
Primary care services with a specialist	4628.3	4641.4	4164.7	6.5 (0.1)	-476.7 (-10.3)	-617.1 to -336.3	<0.001
Primary care services with a NP/PA/CNS	1245.3	1627.7	1625.3	191.2 (15.4)	-2.4 (-0.1)	-153.8 to 148.9	0.975
Primary care services with a FQHC/RHC	469.9	489.4	510.2	9.8 (2.1)	20.8 (4.3)	-110.5 to 152.2	0.756
Skilled nursing facility discharges	65.2	59.7	51.8	-2.8 (-4.2)	-7.9 (-13.2)	-12.9 to -2.9	0.002

While primary care service utilization per 1,000 person years also fell 6.7% (p<0.001), there were important distinctions by the type of primary care provider. Primary care services with a primary care physician or a specialist both declined, while primary care visits with a Nurse Practitioner (NP), Physician Assistant (PA), or Clinical Nurse Specialists (CNS) or with a FQHC/RHC remained flat (Table 2). Importantly, the stability of primary care utilization with a NP, PA, or CNS reflects a dramatic departure from the 15.4% annual growth in utilization of these services per 1,000 person years in prior years (2017 to 2019).

### Changes in quality performance

The average ACO quality score rose 3.2 points (95% CI, 2.9 to 3.4, p<0.001), a result driven by increases in tobacco use screening and intervention (3.4 percentage point increase, 95%CI, 1.5 to 5.2, p<0.001), colorectal screening (1.4 percentage point increase, 95%CI, 0.1 to 2.7, p = 0.04), and statin use (1.0 percentage point increase, 95% CI, 0.0 to 1.8, p = 0.04) as well as a reduction in the rate of hospital discharges for ambulatory-sensitive conditions (95%CI, -0.9 to -0.8, p<0.001) (Table 3). However, the proportion of patients with poor diabetes control and uncontrolled high blood pressure increased from 13.7 to 14.7% (95% CI, 0.3 to 1.7, p = 0.008) and from 24.7 to 27.1% (95%CI, -3.3 to -1.5, p<0.001), respectively (Table 3).

**Table 3:** Changes in quality scores, 2017–2020.

Quality Measure:	2017 (n = 472)	2019 (n = 680)	2020 (n = 513)	Annual Change 2017–19 (% Change)	Annual Change 2019–20		
					Annual Change (% Change)	95% CI	p value
Quality Score, out of 100	92.4	94.6	97.8	1.1 (1.2)	3.2 (3.4)	2.9 to 3.4	<0.001
Falls: Screening for Future Fall Risk, %	-- ^a^	84.6	85.0	--	0.4 (0.5)	-1.2 to 1.9	0.636
Preventive Care and Screening: Influenza Immunization, %	-- ^b^	75.1	76.0	--	0.9 (1.2)	-0.5 to 2.2	0.193
Preventive Care and Screening: Tobacco Use: Screening and Cessation Intervention, %	-- ^c^	78.3	81.7	--	3.4 (4.3)	1.5 to 5.2	<0.001
Preventive Care and Screening: Screening for Depression and Follow-Up Plan, %	62.0	71.1	71.5	4.6 (7.3)	0.4 (0.6)	-1.6 to 2.3	0.723
Colorectal Cancer Screening, %	64.7	71.2	72.6	3.3 (5.0)	1.4 (2.0)	0.1 to 2.7	0.041
Breast Cancer Screening, %	70.2	74.2	74.0	2.0 (2.8)	-0.2 (-0.3)	-1.3 to 1.1	0.824
Diabetes: Hemoglobin A1c (HbA1c) Poor Control (>9%), %	16.7	13.7	14.7	-1.5 (-9.0)	1.0 (7.3)	0.3 to 1.7	0.008
Controlling High Blood Pressure, %	71.7	75.3	72.9	1.8 (2.5)	-2.4 (-3.2)	-3.3 to -1.5	<0.001
Depression Remission at Twelve Months, %	8.2	13.7	14.0	2.8 (33.5)	0.3 (2.2)	-1.2 to 1.8	0.688
Statin Therapy for the Prevention and Treatment of Cardiovascular Disease, %	79.9	82.4	83.4	1.3 (1.6)	1.0 (1.2)	0.0 to 1.8	0.038
Ambulatory Sensitive Condition Acute Composite, per 100 person years	1.9	1.9	1.0	0.0 (-0.8)	-1.0 (-50.0)	-0.9 to -0.8	<0.001

^a^ACO13 was not an included quality measure in MSSP in 2017.

^b^ACO14 was not an included quality measure in MSSP in 2017.

^c^ACO17 was not an included quality measure in MSSP in 2017.

In sensitivity analysis analyzing only ACOs in existence in both 2019 and 2020, there were no substantial differences in findings from the main analysis (S1–S3 Tables). A weakly significant increase in the quality measure for statin therapy in the main analysis (95%CI, 0.0 to 1.8, p = 0.038) became insignificant in the sensitivity analysis (95%CI, 0.2 to 1.6, p = 0.112) (S3 Table).

## Discussion

In a national study of Medicare ACOs, we find that the COVID-19 pandemic led to substantial decreases in non-COVID spending and utilization among Medicare beneficiaries in MSSP. Despite this, ACOs did not experience a drop in overall quality score and in fact improved on measures of preventive screening for certain conditions. However, quality measures for chronic conditions like diabetes and hypertension worsened, which raises concern.

To our knowledge, this study provides the most comprehensive analysis to date of changes in spending, utilization, and quality in MSSP from before to after the start of the COVID pandemic. Our study adds granularity and detail to the literature by highlighting specific areas in which cost, quality, and utilization changed. In terms of spending, the 8.3 percent drop in per capita non-COVID spending propelled the program to a year of record net savings of $1.9 billion compared to benchmark, with 67% of ACOs receiving shared savings bonuses [3]. Whether these per capita spending reductions in both inpatient and outpatient spending will hold in 2021 and beyond remains a key question. The unprecedented period of cancelled elective surgeries and delay of non-urgent care early in the pandemic did not repeat to such a degree in 2021 [17, 18]. By the end of 2020, outpatient visits had returned to pre-pandemic levels [19].

With utilization, MSSP measures mirrored national trends in some categories and bucked the trend in others. Decreases in non-COVID hospitalizations in MSSP ACOs aligns with an approximately 8.9% drop in hospitalizations nationally in 2020 [20]. Outpatient visits and preventive service utilization, such as cancer screenings, also fell nationally in 2020 [19, 20]. Although no change in per capita post-acute care spending was noted from 2019 to 2020, participation in MSSP was previously found to be associated with reduction in spending in this category [21]. However, MSSP appears to have outperformed the national average in preventive screenings, which remained flat or even increased (as with tobacco use screening and colorectal cancer screening) among beneficiaries aligned with MSSP. This suggests that tying financial incentives to quality performance helps sustain or improve rates of preventive service utilization even during a pandemic, wherein related restrictions hinder access to care. For instance, anecdotal reports suggest that at least some ACOs adapted on the colorectal cancer screening metric by shifting to home-based fecal occult blood tests and fecal immunochemical tests as opposed to flexible sigmoidoscopy or colonoscopy [22]. The increases in preventive service use in MSSP despite the COVID pandemic are particularly important in context of an earlier study finding that MSSP program exit is associated with a reduction in preventive service use. Together, these data add further evidence that tying financial incentives to quality performance can affect provider behavior [23].

These improvements, or absence of worsening, in screening rates helped increase the average quality score of ACOs in 2020. However, direct comparison of the 2019 and 2020 quality scores is complicated by differences in assessed metrics as described in the methods. In addition, the worsening rates of diabetes and blood pressure control in 2020 raise concerns that compromised chronic disease management will lead to worsened health and preventable cost and utilization for many years to come [24, 25]. It also suggests that while ACOs offer lessons in maintaining rates of screenings, ACO and non-ACO providers alike ought to explore innovative strategies to manage chronic disease in this transformed clinical practice environment. Importantly, efforts to improve chronic disease management should experiment beyond traditional care management and coordination techniques seeing as prior evaluations suggest their limited impact on improving spending and quality outcomes [26, 27].

Our findings have several policy implications. First, despite initial fears of severe financial losses, these data indicate that with CMS’s COVID flexibilities, MSSP experienced financial success against benchmark and quality performance on a reduced set of metrics [2, 10, 28, 29]. Second, as COVID approaches endemicity, the question arises whether excluding COVID-related costs from benchmark calculation remains appropriate in future years. The exclusion almost certainly facilitated ACOs’ ability to meet benchmark and earn nearly $2.3 billion in shared savings in 2020. As these shared savings reflect taxpayer dollars, it is essential that they are spent rewarding true success in improving care delivery as opposed to shifts in care utilization related to COVID care. Third, the worsening indicators of chronic disease management in 2020 suggest that a rise in chronic disease-related complications is upon us with implications for higher Medicare spending and utilization. As such, a renewed focus on primary care and chronic disease management is especially important, and financing reform programs offer vehicles for CMS to further incentivize these activities. Additionally, the worsening chronic disease measures suggest that savings achieved by ACOs in 2020 may have come in substantial part from foregone necessary care as opposed to improved efficiency and elimination of low-value care.

## Conclusion

The MSSP experienced substantial changes in healthcare service use related to the COVID-19 pandemic, leading to record savings for ACOs participating in MSSP. However, additional study is needed to understand the extent to which this reflects elimination of low-value care versus foregone necessary care. Quality performance suggests that ACOs outperformed the national average on preventive screenings but, like other providers, struggled to prevent worsening of chronic metabolic diseases. Additionally, the appropriateness of the benchmark methodology and exclusion of COVID-related spending, especially as the virus approaches endemicity, should be revisited to ensure bonus payments reflect advances in care delivery and health outcomes rather than pandemic-related shifts in spending and utilization. Future research, using more granular, provider-level data, could contribute to this effort by examining geographic variation in ACO performance during COVID as well as study the impacts of long-term COVID health outcomes and other common chronic conditions.

## Supporting information

S1 Table Sensitivity analysis with only ACOs in both 2019 and 2020. (DOCX)

S2 Table Utilization sensitivity analysis with only ACOs in both 2019 and 2020. (DOCX)

S3 Table Quality sensitivity analysis with only ACOs in both 2019 and 2020. (DOCX)
