# Supplementary material for: Changes in spending, utilization, and quality of care among Medicare accountable care organizations during the COVID-19 pandemic
Source: PLoS One. 2022 Aug 12;17(8):e0272706. doi: 10.1371/journal.pone.0272706 (PMC9374212; doi:10.1371/journal.pone.0272706)
Supplement: S1 Table — (DOCX) [file pone.0272706.s001.docx]

**S1 Table. Sensitivity Analysis with only ACOs in both 2019 and 2020.**

| **Per Capita Expenditure** | **2019 Per Capita Expenditures (n = 596)** | **2020 Per Capita Expenditures (n = 460)** | **Annual Change, 2019-20** | | |
| --- | --- | --- | --- | --- | --- |
|  |  |  | **% Change in Per Capita Expenditure** | **95% CI** | **p value** |
| Total | 11522.1 | 10476.0 | -9.1 | 11018.9 to 11293.5 | <0.001 |
| **Acute Inpatient Care** | **3085.2** | **2628.3** | **-14.8** | **-542.8 to -371.1** | **<0.001** |
| Short Term Acute Care Hospital | 3017.5 | 2561.4 | -15.1 | -531.7 to -380.4 | <0.001 |
| Inpatient Psychiatric Hospital | 79.4 | 66.9 | -15.7 | -21.4 to -3.7 | 0.005 |
| Other Inpatient | 3.6 | 0.0 | -100.0 | -5.6 to -1.7 | <0.001 |
| **Post-Acute Care** | **2040.5** | **1931.8** | **-5.3** | **-245.6 to 27.9** | **0.119** |
| Long Term Care Hospital | 89.6 | 74.6 | -16.7 | -24.9 to -5.3 | 0.003 |
| Inpatient Rehabilitation Facility | 282.0 | 267.9 | -5.0 | -33.5 to 5.2 | 0.153 |
| Hospice | 296.0 | 305.5 | 3.2 | -25.9 to 44.9 | 0.599 |
| Skilled Nursing Facility or Unit | 789.6 | 736.8 | -6.7 | -136.2 to 30.6 | 0.214 |
| Home Health | 583.2 | 547.0 | -6.2 | -73.7 to 1.4 | 0.059 |
| **Total Outpatient** | **6295.0** | **5802.6** | **-7.8** | **-598.6 to -386.1** | **<0.001** |
| Outpatient | 2587.1 | 2429.4 | -6.1 | -254.5 to -60.9 | 0.001 |
| Physician/Supplier | 3707.9 | 3373.2 | -9.0 | -443.3 to -226.1 | <0.001 |
| Durable Medical Equipment | 293.1 | 300.9 | 2.7 | -0.9 to 16.4 | 0.081 |
| Ambulance | 141.8 | 123.3 | -13.0 | -27.9 to -8.9 | <0.001 |
