# Supplementary material for: Changes in spending, utilization, and quality of care among Medicare accountable care organizations during the COVID-19 pandemic
Source: PLoS One. 2022 Aug 12;17(8):e0272706. doi: 10.1371/journal.pone.0272706 (PMC9374212; doi:10.1371/journal.pone.0272706)
Supplement: S2 Table — (DOCX) [file pone.0272706.s002.docx]

**S2 Table. Utilization Sensitivity Analysis with only ACOs in both 2019 and 2020.**

| **Utilization Measure (per 1000 person years):** | **2019 Utilization (n = 596)** | **2020 Utilization (n = 460)** | **Annual Change, 2019-20** | | | |
| --- | --- | --- | --- | --- | --- | --- |
|  |  |  | **Change in Utilization** | **% Change in Utilization** | **95% CI** | **p Value** |
| Inpatient Hospital Discharges | 312.4 | 267.5 | -44.9 | -14.4 | -52.6 to -37.2 | <0.001 |
| Short term acute care hospital discharges | 287.8 | 246.0 | -41.8 | -14.5 | -48.7 to -34.9 | <0.001 |
| Long term care hospital discharges | 2.5 | 2.3 | -0.2 | -8.0 | -0.6 to 0.1 | 0.141 |
| Inpatient Rehab Facility discharges | 14.2 | 13.0 | -1.2 | -8.5 | -2.2 to -0.1 | 0.026 |
| Inpatient Psychiatric Facility Discharges | 7.7 | 6.0 | -1.7 | -22.1 | -2.4 to -0.9 | <0.001 |
| Congestive Heart Failure Discharges | 16.8 | 13.5 | -3.3 | -19.6 | -3.6 to -2.6 | <0.001 |
| COPD or Asthma discharges | 7.8 | 4.5 | -3.3 | -42.3 | -3.7 to -3.0 | <0.001 |
| Post-discharge provider visits (30 day) | 808.7 | 779.0 | -29.7 | -3.7 | -33.9 to -25.4 | <0.001 |
| Outpatient ED visits | 713.6 | 578.0 | -135.6 | -19.0 | -152.7 to -118.6 | <0.001 |
| Inpatient ED Visits | 217.2 | 191.5 | -25.7 | -11.8 | -33.1 to -18.3 | <0.001 |
| CT Events | 710.8 | 647.4 | -63.4 | -8.9 | -76.4 to -50.2 | <0.001 |
| MRI Events | 244.7 | 217.5 | -27.2 | -11.1 | -33.7 to -20.8 | <0.001 |
| Primary Care Services | 10973.1 | 10166.1 | -807 | -7.4 | -1100.7 to -513.4 | <0.001 |
| Primary Care Services with PCP | 4181.6 | 3892.5 | -289.1 | -6.9 | -0.6 to 0.1 | 0.003 |
| Primary Care services with a specialist | 4637.1 | 4150.2 | -486.9 | -10.5 | -635.4 to -338.3 | <0.001 |
| Primary Care services with a NP/PA/CNS | 1646.4 | 1619.4 | -27 | -1.6 | -191.8 to 137.8 | 0.784 |
| Primary Care services with a FQHC/RHC | 508.0 | 502.9 | -5.1 | -1.0 | -146.4 to 138.1 | 0.955 |
| Skilled nursing facility discharges | 60.4 | 51.3 | -9.1 | -15.1 | -14.4 to -3.7 | 0.001 |
