# Supplementary material for: Changes in spending, utilization, and quality of care among Medicare accountable care organizations during the COVID-19 pandemic
Source: PLoS One. 2022 Aug 12;17(8):e0272706. doi: 10.1371/journal.pone.0272706 (PMC9374212; doi:10.1371/journal.pone.0272706)
Supplement: S3 Table — (DOCX) [file pone.0272706.s003.docx]

**S3 Table. Quality Sensitivity Analysis with only ACOs in both 2019 and 2020.**

| **Quality Measure:** | **2019 Quality Score**  **(n = 596)** | **2020 Quality Score**  **(n = 460)** | **Annual Change, 2019-20** | | | |
| --- | --- | --- | --- | --- | --- | --- |
|  |  |  | **Change in Quality** | **% Change in Quality Score** | **95% CI** | **p Value** |
| Quality Score, out of 100 | 94.8 | 97.7 | 2.9 | 3.1 | 2.7 to 3.1 | <0.001 |
| Falls: Screening for Future Fall Risk, % | 84.9 | 85.3 | 0.4 | 0.5 | -1.2 to 2.1 | 0.617 |
| Preventive Care and Screening: Influenza Immunization, % | 75.6 | 76.4 | 0.8 | 1.1 | -0.6 to 2.3 | 0.232 |
| Preventive Care and Screening: Tobacco Use: Screening and Cessation Intervention, % | 78.7 | 81.9 | 3.2 | 4.1 | 1.4 to 5.2 | 0.001 |
| Preventive Care and Screening: Screening for Depression and Follow-Up Plan , % | 71.8 | 71.4 | -0.4 | -0.6 | -2.5 to 1.6 | 0.627 |
| Colorectal Cancer Screening , % | 71.7 | 73.1 | 1.4 | 2.0 | 0.1 to 2.8 | 0.040 |
| Breast Cancer Screening, % | 74.7 | 74.4 | -0.3 | -0.4 | -1.5 to 1.0 | 0.714 |
| Diabetes: Hemoglobin A1c (HbA1c) Poor Control (>9%), % | 13.5 | 14.6 | 1.1 | 8.1 | 0.4 to 1.9 | 0.003 |
| Controlling High Blood Pressure, % | 75.5 | 72.8 | -2.7 | -3.6 | -3.7 to -1.7 | <0.001 |
| Depression Remission at Twelve Months, % | 13.8 | 14.5 | 0.7 | 5.1 | -0.9 to 2.3 | 0.412 |
| Statin Therapy for the Prevention and Treatment of Cardiovascular Disease, % | 82.7 | 83.5 | 0.8 | 1.0 | 0.2 to 1.6 | 0.112 |
| Ambulatory Sensitive Condition Acute Composite, per 100 person years | 1.9 | 0.9 | -1 | -52.6 | -0.9 to -0.8 | <0.001 |
